# Gene Editing for Inherited Red Blood Cell Diseases

**DOI:** 10.3389/fphys.2022.848261

**Published:** 2022-03-28

**Authors:** Oscar Quintana-Bustamante, Sara Fañanas-Baquero, Mercedes Dessy-Rodriguez, Isabel Ojeda-Pérez, Jose-Carlos Segovia

**Affiliations:** ^1^Hematopoietic Innovative Therapies Division, Centro de Investigaciones Energéticas, Medioambientales y Tecnológicas (CIEMAT) and Centro de Investigación Biomédica en Red de Enfermedades Raras (CIBERER), Madrid, Spain; ^2^Instituto de Investigación Sanitaria Fundación Jiménez Díaz (IIS-FJD, UAM), Unidad Mixta de Terapias Avanzadas, Madrid, Spain

**Keywords:** gene therapy (GT), gene editing, hemolytic anemias, pyruvate kinase deficiency (PKD), hemoglobinopathies

## Abstract

Today gene therapy is a real therapeutic option to address inherited hematological diseases that could be beneficial for thousands of patients worldwide. Currently, gene therapy is used to treat different monogenic hematological pathologies, including several red blood cell diseases such as β-thalassemia, sickle cell disease and pyruvate kinase deficiency. This approach is based on addition gene therapy, which consists of the correction of hematopoietic stem cells (HSCs) using lentiviral vectors, which integrate a corrected version of the altered gene. Lentivirally-corrected HSCs generate healthy cells that compensate for the deficiency caused by genetic mutations. Despite its successful results, this approach lacks both control of the integration of the transgene into the genome and endogenous regulation of the therapeutic gene, both of which are important aspects that might be a cause for concern. To overcome these limitations, gene editing is able to correct the altered gene through more precise and safer approaches. Cheap and easy-to-design gene editing tools, such as the CRISPR/Cas9 system, allow the specific correction of the altered gene without affecting the rest of the genome. Inherited erythroid diseases, such as thalassemia, sickle cell disease and Pyruvate Kinase Deficiency, have been the test bed for these gene editing strategies, and promising results are currently being seen. CRISPR/Cas9 system has been successfully used to manipulate globin regulation to re-activate fetal globin chains in adult red blood cells and to compensate for hemoglobin defects. Knock-in at the mutated locus to express the therapeutic gene under the endogenous gene regulatory region has also been accomplished successfully. Thanks to the lessons learned from previous lentiviral gene therapy research and trials, gene editing for red blood cell diseases is rapidly moving from its proof-of-concept to its first exciting results in the clinic. Indeed, patients suffering from β-thalassemia and sickle cell disease have already been successfully treated with gene editing, which will hopefully inspire the use of gene editing to cure erythroid disorders and many other inherited diseases in the near future.

## Introduction

Anemia is a condition in which the number of red blood cells (RBCs), and consequently their oxygen-carrying capacity, is insufficient to meet the body’s physiological needs. Anemia can occur due to reduced or impaired erythropoiesis, increased RBC destruction, or blood loss (WHO, 2015). Anemia derived symptoms result from impaired tissue oxygen delivery or as a consequence of iron overload caused by the massive destruction of RBCs. Erythrocyte reduction impairs oxygen transport. Consequently, it may cause weakness, fatigue, difficulty concentrating, and/or poor work performance. In addition, iron overload may cause chronic liver diseases and cirrhosis heart failure among other derived pathologies. Although the top cause of anemia globally is iron-deficiency, there are other leading causes of anemia include inherited disorders such as hemolytic anemias that result from increased RBC destruction (Cazzola, 2020). Among these congenital RBC diseases, hemoglobinopathies have the highest incidence. Sickle Cell Disease (SCD) incidence is especially high, since about new 300,000 SCD patients are born every year^1^, mainly in Africa and India. This is a recessive genetic disorder caused by the sickling mutation in the β-globin gene, an amino acid change from a polar residue, glutamic acid (E), to a hydrophobic one, valine (V), commonly referred as “E6V.” SCD signs include a low number of red blood cells (anemia), repeated infections, and painful veno-oclussive episodes, caused when sickled red blood cells get stuck in small blood vessels.

**FIGURE 1:**
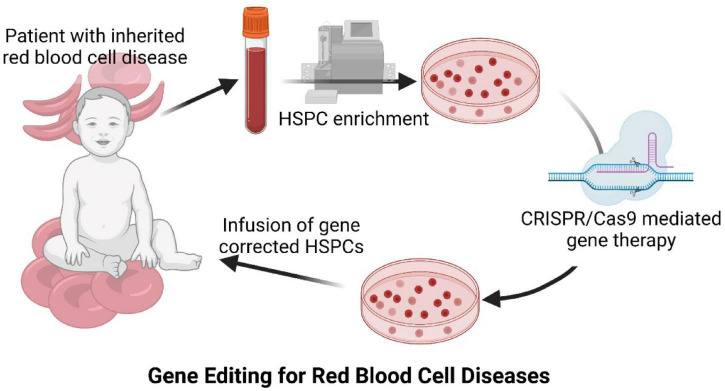
Diagram of CRISPR/Cas9 mediated gene therapy approach for inherited red blood cell diseases.

Despite the high number of patients affected by inherited anemias, the only curative treatment for severe patients suffering from inherited RBC diseases is restricted to allogeneic Hematopoietic Stem Cell Transplantation (HSCT), which has particularly serious side effects in these patients. However, over the last few years, new and exciting therapeutic options have emerged for this group of diseases, as well as for other inherited hematopoietic pathologies. The implementation of new diagnostic technologies, such as next-generation sequencing (Kaestner and Bianchi, 2020), together with the development of new therapeutic approaches such as gene therapy (Bueren et al., 2020), have considerably improved the knowledge of inherited anemias and have paved the way to their definitive cure. Nowadays, these two fields have coincided to allow the identification of the specific genetic alterations that cause the different inherited anemias, thus allowing the development of new gene therapy strategies [reviewed by Ferrari et al. (2021)]. Gene therapy facilitates the treatment of monogenic blood diseases through autologous HSCT of gene corrected Hematopoietic Stem and Progenitor Cells (HSPCs), where the low number of HLA compatible HSPC donors and the risk of suffering graft-versus host disease (GvHD) hamper allogeneic HSCT use. HSPCs are purified from the patients, then gene corrected *ex vivo* before being re-infused into the patients. Autologous gene corrected HSPCs engraft into the patient’s bone marrow niche, start to proliferate and differentiate to reconstitute a corrected hematopoiesis (Figure 1). These corrected HSPCs have the potential for lifelong maintenance of the patient’s hematopoietic system. Therefore, all the new hematopoietic cells, including RBCs, are derived from corrected HSPCs and carry the genetic correction to restore the normal functions of the gene mutated in the inherited disease. Hemoglobinopathies have been the first inherited red blood diseases to be addressed by gene therapy because of the number of affected patients and the deep knowledge about their genetic causes.

Addition (by means of lentiviral vector correction) and gene editing therapy are two alternatives to address hematopoietic inherited diseases *via* gene therapy. In this review, we will first briefly explain addition gene therapy, and then focus on the treatment of inherited RBC disorders through gene editing. Gene therapy based on gene editing will be addressed from basic research to the clinical application for the correction of inherited RBC diseases.

## Additional Gene Therapy: Current Successes

Autologous HSCT of genetically corrected cells will mean the definitive treatment for inherited anemias, which will overcome the limitations of allogeneic HSCT, such as limited availability of HLA-matched donors, infections linked to immunosuppression or development of GvHD. Viral vectors based on the family *Retroviridae* were selected to deliver the therapeutic genetic materials due to their ability to integrate into the host cell genome, which facilitates the correction of the transduced cells and all their progeny (Belmont et al., 1986; Hock and Miller, 1986; Kantoff et al., 1986; Keller and Wagner, 1986; Dzierzak et al., 1988; Hughes et al., 1989; Lim et al., 1989; Szilvassy et al., 1989). This is particularly important in the case of highly proliferative tissues with a small number of stem cells responsible for the maintenance of the whole system, such as in the hematopoietic system. In these systems, the genetic correction of the stem cell population will be transmitted to all different and functional mature cells. Seminal papers showed the feasibility of this strategy using gamma-retroviral vectors (Williams et al., 1984) and the first clinical trials demonstrated their clinical applicability (Dunbar and Kohn, 1996; Abonour et al., 2000; Cavazzana-Calvo et al., 2000; Aiuti et al., 2002; Hacein-Bey-Abina et al., 2002).

However, one of the best clinical successes of gene therapy, the gene therapy cure of X-linked severe combined immunodeficiency (X-SCID) patients, turned out to be a major bottleneck in the field because few patients developed leukemia caused by gamma-retroviral insertional mutagenesis (Hacein-Bey-Abina et al., 2003; Howe et al., 2008). Additional basic research in new vector backbones (lentiviral vectors) and new regulatory sequences (endogenous and less potent promoters) helped to move permanent gene therapy forward again. Nowadays, lentiviral gene therapy is the preferred option to genetically correct HSPCs. More than 350 patients with different pathologies have been successfully treated with this strategy. Many inherited hematological diseases (Bueren et al., 2020; Ferrari et al., 2021), including hemoglobinopathies like β-thalassemia (Cavazzana-calvo et al., 2010; Thompson et al., 2018; Locatelli et al., 2021; Magrin et al., 2022) or SCD (Holmes et al., 2017; Ribeil et al., 2017; Kanter et al., 2021; Magrin et al., 2022), have been addressed (Figure 1). In fact, lentivirally corrected HSPCs have been approved for clinical application in transfusion-Dependent β-thalassemia patients (Zynteglo^2^). β-Thalassemia occurs when an abnormal form or inadequate amount of hemoglobin is produced due to mutations in the *HBB* gene. HBB mutations cause a reduced or absent synthesis of the hemoglobin beta chains that results in variable outcomes ranging from severe anemia to clinically asymptomatic individuals. Global annual incidence is estimated at one in 100,000. Affected individuals also suffer from anemia, which can cause pale skin, weakness, fatigue, and more serious complications. As we have focused on inherited hemolytic anemias, we have recently developed a lentiviral vector to genetically correct Pyruvate Kinase Deficiency (PKD) (Garcia-Gomez et al., 2016). PKD is an autosomal-recessive disorder caused by mutations in the pyruvate kinase gene (*PKLR*) which encodes for the erythroid pyruvate kinase protein (RPK). RPK is responsible for the maintenance of normal erythrocyte ATP levels. The disease becomes clinically relevant when protein activity decreases below 25% of the normal activity in erythrocytes. Most frequent clinical signs are mild to very severe anemia, reticulocytosis, splenomegaly, and iron overload, which may be life-threatening in severely affected patients. PKD is considered the most common cause of chronic non-spherocytic hemolytic anemia (CNSHA), with an estimated prevalence of about 1:20,000. A phase I lentiviral gene therapy clinical trial is ongoing to assess its safety and efficacy (NCT04105166). Preliminary data from the first two PKD patients transplanted with lentiviral-corrected autologous HSPCs have shown a fast hematologic recovery after transplant (13 days) and a complete recovery of erythroid parameters more than 18 months post-transplant (Shah et al., 2021), thus demonstrating the feasibility of this strategy to treat hemolytic anemias as well (Figure 1).

Unfortunately, uncontrolled cell proliferation resulting in pre-leukemic or leukemic events has been recently reported in patients transplanted with lentivirally corrected T cells (Fraietta et al., 2018) and HSPCs (Jofra Hernández et al., 2021). This point should be carefully studied in the case of patients affected with hemoglobinopathies, especially SCD, and treated with lentiviral gene therapy (Goyal et al., 2021). Although the number of adverse events is very low, there are still important challenges to offering the safest and most efficacious gene therapy. Among others, the most relevant are the elimination of insertional oncogenesis and the endogenous regulation of the therapeutic gene.

## Gene Editing Technology

The initial concepts of directed gene modification, or gene editing, arose during the 1960s and early 1970s when restriction enzymes were used to manipulate DNA (Smith and Wilcox, 1970; Scherer and Davis, 1979) which created specific cuts in the DNA. In the following decade, Capecchi demonstrated that new DNA sequences could be introduced into the genome by homologous recombination (HR), which allows the transfer of any modification of into the genome of a living cell (Capecchi, 1989). In 1985, Smithies introduced targeted genomic changes in the context of a blood disorder, with the insertion of a plasmid DNA sequence into the human chromosomal β-globin locus to correct harmful mutations in patients with thalassemia and SCD (Smithies et al., 1985). However, HR efficiency was very limited in eukaryotic cells. The solution to this constraint appeared in the late 1980s and early 1990s, when it was found that the induction of a double strand break (DSB) enhanced HR at a specific genomic target efficiently (Rudin et al., 1989; Rouet et al., 1994). A DSB triggers the cell’s endogenous DNA repair mechanisms to the site where the cut was induced. Different mechanisms apply. Nonhomologous end joining (NHEJ) and microhomology-mediated end joining (MMEJ) involve modifying the two broken ends without a template to make then compatible to rejoin (Lieber et al., 2003; Guirouilh-Barbat et al., 2004). NHEJ directly ligates the broken ends without any template. However, MMEJ repairs the broken ends through an alignment of microhomology sequences, which are produced by end resection (San Filippo et al., 2008; Truong et al., 2013; Chang et al., 2017; Pickar-Oliver and Gersbach, 2019). In HR the broken strand relies on a repair in a template-directed manner (Szostak et al., 1983). Various endonuclease-based mechanisms to introduce DSBs with high precision have been developed. All the different types of endonucleases are capable of precisely modifying genomic DNA, delete, insert or even replace DNA fragments in a highly targeted and selected way. The most-used endonucleases will be described below.

### Meganucleases

Meganucleases are DNA nucleases that have the ability to cleave dsDNA at specific recognition sites. The main advantage of this technology are the high specificity to target DNA due to large recognition sites [14–40 base pairs (bp)]. Meganucleases include enzymes such as I-SceI and I-CreI, which can be used to target genes in mammalian cells (Cohen-Tannoudji et al., 1998; Grizot et al., 2010). Although there are many available natural meganucleases, the probability of finding an enzyme that targets a desired locus is very small, which limits their use (Fenina et al., 2012). Molecular engineering processes have been developed that allow the design of engineered meganucleases. However, production of customized meganucleases remains complex and highly inefficient, limiting its broad application.

### Zinc Finger Nucleases

Zinc finger nucleases (ZFNs) can be engineered to target specific DNA sequences. Chandrasegaran’s group in 1996 reported the generation of the first ZFN by fusing zinc finger protein motifs (that confer the DNA binding selectivity) to the cleavage domain of the FokI endonuclease (Kim et al., 1996). In 2003, Baltimore and Porteus demonstrated that ZFN enhanced gene targeting in human cell lines (Porteus and Baltimore, 2003). The first clinical trial using ZFN focused on the disruption of CCR5 (the HIV co-receptor) (Tebas et al., 2014). ZFNs have been used to disrupt the erythroid enhancer of the *BCL11A* gene, which is a repressor of γ-globins in adult erythrocytes, in HSPCs to increase fetal globin expression and reduce sickling in erythroid cells as gene therapy for SCD patients (Holmes et al., 2017). This approach is currently being assessed in two phase 1/2a clinical trials for transfusion-dependent β-thalassemia (NCT03432364) and SCD (NCT03653247), which will be described below. However, ZFN toxicity, because of cleavage at undesired genomic locations (Porteus and Baltimore, 2003), and its highly complicated design (Bae et al., 2003; Maeder et al., 2008) hamper its wider use.

### Transcription Activator-Like Effector Nucleases

To overcome the limitations of ZFNs, a new series of nucleases, called transcription activator-like effector nucleases (TALENs), were developed (Christian et al., 2010; Li et al., 2011), Similar to ZFNs, TALENs are engineered restriction enzymes that are fused to the catalytic domains of the FokI endonuclease and shown to function as dimers to cleave a DNA target site. This system has been shown to efficiently target genomes in both human somatic cells (Miller et al., 2011) and human pluripotent stem cells (Hockemeyer et al., 2011). The therapeutic potential of TALEN technology was also demonstrated with the publication of a gene edited Chimeric Antigen Receptor T cells (CART cells) clinical trial to treat B cell acute lymphoblastic leukemia (Qasim et al., 2017). More recently, TALEN technology has also been applied to RBC inherited diseases, such as hemoglobinopathies (Lux et al., 2019) and PKD (Garate et al., 2015; Quintana-Bustamante et al., 2019).

### CRISPR/Cas System

CRISPR/Cas system [Clustered Regularly-Interspaced Short Palindromic Repeats and Cas (CRISPR associated) nucleases] is the most recently-developed platform in the field of genome editing. CRISPR/Cas system has changed the gene editing landscape. This newcomer has broadened the potential therapeutic applications of gene editing to correct inherited diseases. CRISPR discovery began in bacteria in 1993 (Mojica et al., 1993). However, it was not until 2012 that this system was developed as a genomic editing tool (Jinek et al., 2012). DNA recognition of the CRISPR/Cas system is based on RNA-DNA interactions. The CRISPR/Cas9 system is made of Cas9 nuclease and single-guide RNA (sgRNA). The sgRNA is an engineered single RNA molecule containing crispr RNA (crRNA) and tracr RNA parts. The sgRNA recognizes the target sequence by standard Watson-Crick base pairing. It has to be followed by a DNA motif called a protospacer adjacent motif (PAM). This sequence is located directly downstream of the target sequence in the genomic DNA, on the non-target strand. The Cas9 nuclease recognizes this interaction and then performs the DNA cleavage, which results in DSB.

CRISPR/Cas9 system is widely used due to its simplicity and versatility. CRISPR/Cas systems have the therapeutic potential to genetically correct inherited RBC diseases, mainly hemoglobinopathies, which have been the workbench for trying new gene editing approaches and will be discussed further in the text. Briefly, CRISPR/Cas9 system has been used to re-express fetal globins, either by recreating hereditary persistence of fetal hemoglobin (HPFH) (Ye et al., 2016) or by knock-out of the fetal globin repressor *BCL11A* (Canver et al., 2015; Brendel et al., 2016; Wu et al., 2019). This gene editing strategy as one of the most promising therapeutic alternatives for hemoglobinopathies. This last strategy has already shown clinical benefit in the correction of β-thalassemia and SCD in patients (Frangoul et al., 2021). Moreover, the mutated *HBB* gene has been gene edited to recover a fully functional β-globin protein through the correction of sickle cell mutation (Dever et al., 2016; DeWitt et al., 2016; Hoban et al., 2016; Lattanzi et al., 2021; Wilkinson et al., 2021). Additionally, another inherited erythroid disease, PKD, has been proposed to be corrected through CRISPR/Cas gene editing (Fañanas-Baquero et al., 2021).

Today, new CRISPR/Cas systems have been described and adapted for gene editing. Different variations of the original CRISPR/Cas9 system have emerged to expand the gene editing toolbox. CRISPR-Associated transposases (Klompe et al., 2019), base editors (Komor et al., 2016; Gaudelli et al., 2017) and prime editing (Anzalone et al., 2019), have used CRISPR/Cas9 system targeting property to get a more precise gene modification (Li et al., 2021; Newby and Liu, 2021). In the coming years, the diversification of CRISPR/Cas systems will facilitate new approaches to correct these inherited disorder and others.

## Delivery of Gene Editing Tools

An ideal vector should deliver the gene editing components to a specific cell type, accommodate foreign genes of sufficient size, achieve the level and duration of transgenic expression sufficient to correct the defect and be non-immunogenic. The main obstacle for considering gene editing as a broad therapeutic option is the availability of a good method to deliver the gene editing tools into the target cell and target tissue. The components of gene editing have to be transferred to the cell/nucleus of interest using either *ex vivo* or *in vivo* strategies. This bottleneck is more important when gene editing of HSPCs is considered. Primitive HSPCs are hard to transfect and refractory to viral vector transduction. Several factors must be considered, including physical barriers (cell membranes, nuclear membranes), or stability of gene editing tools inside the target cells and the possible rejection by the immune system of the host (Charlesworth et al., 2019).

**FIGURE 2:**
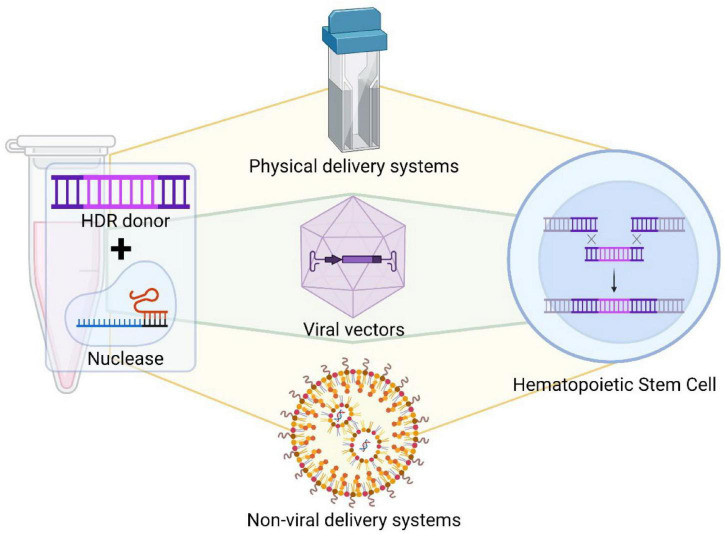
Delivery systems of gene editing tools. Different gene editing tools can be delivered into hematopoietic cells by specific delivery systems, such as physical methods, viral vectors or non-viral delivery system. Each gene editing tool has to be modified to be adapted for a specific delivery method.

Gene delivery strategies can be categorized into three classes: physical delivery, viral vectors, and non-viral agents (Figure 2). Physical delivery of genes can be accomplished by microinjection or electroporation. Microinjection uses a microscope and a 0.5–5.0 μm diameter needle. The cell membrane is pierced and cargoes are delivered directly to a target site within the cell. This process circumnavigates barriers associated with delivery through extracellular matrices, cell membranes, and cytoplasmic components. Either plasmid DNA encoding both the Cas9 protein and the sgRNA, mRNA encoding Cas9 and sgRNA, or Cas9 protein with sgRNA can be directly injected into individual cells (Horii et al., 2014; Nakagawa et al., 2015). However, this strategy requires significant training, it is time consuming and only a very limited number of cells can be manipulated at a time.

Electroporation (Figure 2) utilizes pulsed high-voltage electrical currents to transiently open nanometer sized pores within the cellular membrane of cells, thus allowing components to flow into the cell. Electroporation is less dependent on cell type than other delivery techniques and can efficiently transfer cargo into cells that are traditionally difficult to manipulate. There are many published methods for electroporation of mammalian cells (Dumeau et al., 2019) and it is broadly used nowadays. However, electroporation may cause cell death. To prevent this problem, new electroporation platforms have been designed to increase delivery efficiency and to reduce toxicity. HSPCs can be electroporated effectively and maintain their engraftment and multipotency properties, facilitating the translation of gene editing to the clinic (Peterson et al., 2016).

The second delivery method is based on viruses (Figure 2). Viruses infect cells efficiently to transfer their genetic material into the host without invoking an immune response. This process makes viruses attractive gene therapy vectors. Viral vectors have been engineered to transfer desired genetic material and to make them safer. Viral vectors are also replication incompetent.

Currently, the combination of electroporation for nuclease entry and viral vectors for donor template delivery is the chosen option for some gene editing approaches (Genovese et al., 2014; Dever et al., 2016; De Ravin et al., 2017; Fañanas-Baquero et al., 2021).

Adeno-associated viral vectors (AAV) are the best option of vector to delivery donor templates. AAVs are a single stranded DNA virus that have been extensively utilized for gene therapy. AAVs are not known to cause or be related with any diseases in humans. There is also a wide range of known serotypes which allow for infection of a multitude of cells with different specificities. AAV serotype 6 is able to transduce HSPCs efficiently when it is combined with electroporation (Dever et al., 2016; Charlesworth et al., 2018). Up to 70% of HSPCs can be transduced without altering their engraftment ability. Electroporation of nucleases in combination with rAAV6 to introduce large donor template, has been selected as a very efficient delivery method to gene edit HSPCs for gene therapy of inherited anemias, such as hemoglobinopathies (Dever et al., 2016; Hoban et al., 2016; Lattanzi et al., 2021; Wilkinson et al., 2021) and PKD (Fañanas-Baquero et al., 2021), and other hematopoietic diseases, such as X-linked chronic granulomatous disease (X-CGD) (De Ravin et al., 2017), Wiskott-Aldrich síndrome (WAS) (Rai et al., 2020).

Other viral vector platforms have also been explored, such as adenoviral vectors (HDAdV). Recently, HDAdVs have demonstrated their capacity to deliver base editors *in vivo* to re-express γ-globin in a mouse model carrying a human β-globin locus yeast artificial chromosome (Li et al., 2021). This approach will be discussed below.

Additionally, other non-viral delivery systems have been developed for HSPCs (Figure 2). Although the efficacy to target HSPCs is still low, these systems might overtake delivery platforms based in viral vectors in the near future, as they are easier to produce and more reproducible in their manufacture. These systems would also potentially be lower in price. Currently, different alternatives are being explored. Some of them, based on nanoparticles, have been reported to deliver RNP in order to reactivate fetal globins (Cruz et al., 2021). Recently, Intellia Therapeutics has reported the use of a bone marrow tropic lipid nanoparticles (LNP) able to gene modify HSPCs *in vivo* in a humanized mice model, as a proof-of-concept to treat SCD (Burns, 2021). These non-viral systems are starting to become efficient to transport gene editing tools into HSPC cytoplasm, so they might be easily used for gene editing approaches involving only nucleases, base editors or prime editors, which will be discussed later. Finally, different approaches have been used to increase the stability of the gene editing tools inside the cells, such as chemical modifications (Hendel et al., 2015), addition of alternative *UTR*s (Quintana-Bustamante et al., 2019), or promoting nuclear localization of the nucleases by linking to nuclear localization signal (NLS).

## Gene Editing Strategies

**FIGURE 3:**
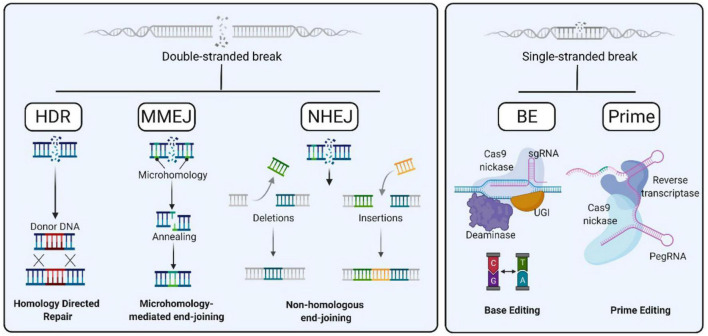
Gene editing strategies. After a Double Strand Break (DSBs) event, the preferential cellular pathways for its repair are homology directed repair (HDR), microhomology mediated end joining (MMEJ), and non-homologous end-joining (NHEJ) **(left)**. DNA can also be repaired in a DSB-independent manner, by means of Base Editing and Prime Editing **(right)**.

Currently, gene editing relies on nucleases. Nucleases were first used to produce DSBs to recruit endogenous DNA repair machinery. Consequently, different gene editing approaches have emerged based on the specific DNA repair mechanism involved: NHEJ, MMEJ and HDR (Figure 3). In addition, alternative strategies based on non-DSB strategies are now being developed, such as base editing and prime editing. In this section, we will describe different gene editing strategies to correct inherited anemias:

### Nonhomologous End Joining-Based Gene Editing

The NHEJ repair pathway is the predominant way to repair DSBs and involves an accumulation of random insertions or deletions (indels) at the cut site (Chang et al., 2017). It is active in all cell cycle phases although it predominantly repairs DSBs during G0 and G1 phases (Shrivastav et al., 2007). This is an error-prone mechanism and usually results in frameshift mutations, often creating premature stop codons and/or a non-functional polypeptide. This pathway has been particularly useful in genetic knock-out experiments and functional genomic CRISPR screens, but it can also be useful in the clinic when gene disruption provides a therapeutic opportunity (Leonova and Gainetdinov, 2020). NHEJ has been widely investigated to correct hemoglobinopathies. As mentioned above, the generation of indels at the *BCL11A* gene, which suppress fetal globins, or at different positions in the β-globin locus, switches adult globin expression to fetal expression in order to compensate for defects of these diseases (Canver et al., 2015; Brendel et al., 2016; Ye et al., 2016; Holmes et al., 2017; Lux et al., 2019; Smith et al., 2019; Frangoul et al., 2021).

The rationale behind this approach is that there are rare asymptomatic SCD patients, some of whom were compound heterozygous for SCD and HPFH mutations. These SCD patients have high levels of fetal hemoglobin (HbF) to compensate their hemoglobinopathy. A hemoglobin molecule is a tetramer formed by two subunits of α-like globin and two subunits of β-like globin. There is a sequential switch of the different β-like globin peptides, from ε-globin in the embryonic hemoglobin, to γ-globin in the fetal hemoglobin of HbF, and then β-globin in the definitive hemoglobin. Several transcription activators and repressors, such as BCL11A, MYB, and KLF1 regulate this transition. With the use of gene editing, the globin switch can be reverted by reproducing mutations such as HPFH to ameliorate the hemoglobinopathy or by eliminating fetal globin repressors such as BCL11A. Canver et al. (2015) led the first attempt to modulate globin switch by inducing indels in the regulatory regions of the *BCL11A* gene. These authors managed to make the reactivation of γ-globin proportional to the level of gene editing of the erythroid enhancer region of *BCL11A* (Canver et al., 2015). Frangoul et al. (2021) have demonstrated the clinical benefit of targeting *BCL11A* with CRISPR/Cas9 system in both SCD and β-thalassemia. Currently, two patients are being treated with this approach and have, one year after the treatment, increases in fetal hemoglobin, transfusion independence and, in the case of SCD, elimination of vaso-occlusive episodes (Frangoul et al., 2021). In addition, other groups have reproduced the HPFH mutation, by TALEN or CRISPR/Cas9 system, as an alternative way to reactivate fetal globins (Ye et al., 2016; Lux et al., 2019).

### Homology Directed Repair-Mediated Knock-In

The other pathway, which is especially appealing to exploit for clinical purposes, is the error-free HR pathway (Figure 3). HR plays an essential role in meiosis and mitosis to exchange genetic information between sister chromatids. It occurs at a relatively low frequency in mammalian cells because it is only active in S-G2 phases in the cell cycle (Shrivastav et al., 2007; San Filippo et al., 2008). This repair pathway needs a homologous DNA template, which can be used to program gene modifications. Whereas NHEJ can be used to produce gene knockouts, HR is useful to introduce specific changes in the target site. This process is called homology directed repair (HDR). To favor this HDR, the exogenous DNA that is intended to be introduced into the genome has to be flanked by homologous arm sequences complementary to the region close to the DSB. HDR has been used to introduce large donor templates, with therapeutic cDNA to compensate the function of the mutated gene, as potential treatment for hemoglobinopathies (Dever et al., 2016) as well as other RBC inherited diseases such as PKD (Fañanas-Baquero et al., 2021).

The introduction of a large therapeutic donor, as mentioned previously, would offer a global solution for all patients, regardless of the nature of the mutation that they carry. Clinically applicable results (up to 70% edited HSPCs) have been obtained using CRISPR/Cas9 system combined with rAAV6 vector in HSPC to correct inherited hematopoietic diseases such as hemoglobinopathies (Dever et al., 2016; Hoban et al., 2016; Lattanzi et al., 2021; Wilkinson et al., 2021), and other hematopoietic diseases such as ase X-CGD (De Ravin et al., 2017), WAS (Rai et al., 2020) and PKD (Fañanas-Baquero et al., 2021). In a seminal paper, Dever et al. (2016) described specific correction of 50% of the sickling mutation in SCD patient-derived HSPCs. They reported a phenotypic correction after differentiation of these HSPCs to erythrocytes by the expression anti-sickling HBB from the cDNA therapeutic donor. These preclinical studies suggest the possibility of using CRISPR-based methodology for targeting HSPCs by HDR at the globin locus, which could be translated into the next generation of therapies for β-hemoglobinopathies. Recently, we have used RNP/rAAV6 platform to treat another red blood disorder such as PKD (Fañanas-Baquero et al., 2021). We described the correction of PKD through precise gene editing at the *PKLR* endogenous locus to keep the tight regulation of the RPK enzyme during erythropoiesis. We have shown efficient correction of the PKD phenotype in patient-derived HSPCs that later differentiate into erythrocytes. Erythroid cells derived from gene edited PKD HSPCs express normal ATP levels (Fañanas-Baquero et al., 2021). This is the proof-of-concept that the metabolic problem characteristic of PKD has been corrected throughout of knock-in of RPK cDNA at *PKLR* gene. This approach will provide a universal therapeutic option for all PKD patients, and the recovery of RPK will be restricted to the erythroid cells without altering the rest of the hematopoietic cells.

It is worth mentioning that estimations regarding the level of correction obtained in the different red blood cell diseases SCD, β-thalassemia and PKD performed at different laboratories are very similar, suggesting that this strategy is reproducible and robust. This would also suggest its maturity could be scaled up to the clinical setting.

### Single-Stranded Oligonucleotides Based Gene Editing

The introduction of a therapeutic donor is one of the limiting steps for the clinical application of gene editing to correct inherited hematopoietic diseases. To overcome this limitation, some groups have pursued a highly specific and personalized approach, with the aim of correcting point mutations of a specific patient with a minimal alteration of his or her gene sequence using nucleases such as CRISPR/Cas9 and single-stranded oligodeoxynucleotides (ssODNs). These single-stranded templates have shown its superiority over similarly designed double-stranded DNA (dsDNA) templates in terms of HDR efficiency, which can be attributed to the use of single-stranded template repair (SSTR), a recently described molecular mechanism for the repair of DNA (Gallagher et al., 2020). Furthermore, the use of ssODNs offers other advantages such as their simple design, short production time and lower cost compared to the use of viral vectors. However, due to limitations in the length of ssDNA that can be synthesized, ssODNs are not currently used to insert long sequences (Miura et al., 2015). Many efforts have been made to determine the optimal characteristics such as symmetry, length of the homology arms or orientations that ssODNs need to display for gene editing purposes but the results are still unclear and locus-dependent (Richardson et al., 2016).

The possible application of this highly specific gene editing system is wide, and some RBC disorders can benefit from it by the *ex-vivo* gene editing of HSPCs, such as is the case of SCD. Different groups have attempted the restoration of *HBB* gene by means of gene correction using different nuclease platforms. Hoban et al. introduced ZFNs, which targeted the SCD causing mutation, as mRNA by electroporation, together with the ssODN that carried the corrective sequence (Hoban et al., 2015). Although the authors were able to detect wild type hemoglobin tetramers in erythroid cells derived from SCD HSPCs, the frequency of correction was very low when tested with HSPCs from healthy donors. Up to 20% gene correction was identified in healthy donor HSPCs by high-throughput DNA sequencing. Once transplanted into immunodeficient mice, gene correction rates below 1% in the bone marrow were obtained. The low efficacy of gene editing in long-term HSPCs hampered its therapeutic application (Hoban et al., 2015). Similarly, DeWitt et al. (2016), Magis et al. (2019) focused on the modification of the E6V region in SCD cells.

Patient-edited cells were able to engraft in immunodeficient mice and around 2.3% gene correction was observed. This 8–10-fold overall decline in efficiencies from *in vitro* results in HSPCs to repopulating *in vivo* results in HSCs is consistent with multiple previous reports observed among the different groups focused on gene editing of HSPCs.

Recently, Pattabhi et al. (2019) directly compared the gene editing outcome of RNP+rAAV6 vs RNP+ssODN in SCD. *In vitro* studies demonstrated that rAAV6 carrying a large donor template promoted greater rates of HDR in contrast to ssODNs. Interestingly, levels of gene correction *in vivo*, analyzed by Next-Generation Sequencing (NGS), were similar in both groups. However, in the analyses performed 12 weeks after transplant, gene correction was lower in the mice receiving cells edited with RNP+rAAV6 (around 0.7%) as compared to mice receiving RNP+ssODN-edited cells (up to 4%). This result is controversial, since Dever et al. (2016) proved much higher gene editing rates when using RNP+rAAV6 for the correction of E6V mutation, even in long-term HSCs (Dever et al., 2016; Lattanzi et al., 2021). Gene editing of β-Thalassemia has also been addressed by ssODN, with similar results to those obtained with SCD (Antony et al., 2018).

Beyond hemoglobinopathies, we have attempted ssODN strategy for the correction of PKD. We have addressed the correction of four mutations in lymphoblastic cell lines derived from two PKD patients, achieving up to 20% gene correction. We have observed reversion of erythrocyte functionality, measured by ATP production, when correcting HSPCs from a PKD patient with ssODNs and differentiating them *in vitro* to erythroid lineage. This suggests CRISPR/Cas9+ssODNs strategy as an additional therapeutically relevant system for PKD patients.

### Use of Non-DSB Strategies: Base Editing/Prime Editing

The latest arrivals in the gene editing arena are base editors (BE) and prime editors (PE) (Figure 3). Both technological developments follow a very clever strategy to recruit proteins able either to convert one nucleotide into another one or to synthesize new DNA sequences *in situ* without requiring dsDNA cleavage or a HDR donor template. These strategies were first developed at Dr. David Liu’s group, who used CRISPR/Cas9 platform to locate DNA modifying enzymes at the target locus. Firstly, base editors were engineered as the fusion between an impaired Cas9 (dCas9 or Cas9 nickase) and cytidine deaminase enzyme in order to directly convert cytosine to uridine, therefore transforming C to T (Komor et al., 2016; Figure 3). A further development in base editing system was made to turn A⋅T to G⋅C through fusing impaired Cas9 with an adenosine deaminase (Gaudelli et al., 2017). This system was called adenine base editor (ABE) to distinguish from the first cytosine base editors (CBE). Together CBEs and ABEs were able to correct most human point mutations causing inherited diseases, since C⋅G to T⋅A transitions account for half of pathogenic point mutations. Different improvements have been made which will increase the efficacy of base editor systems and enhance their accuracy. Despite the good efficacy rate of the latest base editors, the main concerns, as with other gene editing systems, are off-target effects, how to prevent the broad nucleotide interconversion, and how to expand base editor applicability to any genomic sequence. Many efforts are being made to refine base editors (Hu et al., 2018). On the other hand, Prime Editing allows writing new genetic information at a specific target genomic locus by fusing an impaired Cas9 to a reverse transcriptase. Prime editing guide RNA (pegRNA) leads the prime editing system to the desired locus while carrying the genetic information to be inserted (Anzalone et al., 2019). PE allows single nucleotide interconversion and the insertion of few nucleotides in the genomic target with a very high efficiency.

Base editors and prime editors are evolving very quickly from their proof-of-concept to their preclinical application to correct inherited diseases. Base editors have been assessed to correct SCD efficiently (Newby and Liu, 2021). ABE was able to convert the SCD allele into the non-pathogenic Makassar variant. Eighty percent of HSPCs derived from patients exchange the SCD polymorphism (CAC) to CGC present in Makassar β-globin. When the gene edited SCD HSPCs were transplanted into immunodeficient mice, hypoxia-induced sickling was reduced threefold. This preclinical work opens the way to translation of base editors into the clinics. Li et al. have also described how ABE vectors targeting the erythroid *BCL11A* enhancer or recreating naturally occurring HPFH mutations in the *HBG1/2* promoter could reactivate γ-globin in a mouse model through helper-dependent adenovirus vectors expressing BEs. Moreover, *in vivo* use of base editing or prime editing could be easily achievable, as it has been shown to correct Hutchinson-Gilford progeria syndrome (Koblan et al., 2021) or β-hemoglobinopathies (Newby and Liu, 2021). Future clinical application might be sped up by the fact that these new gene editing tools do not require dsDNA cleavage or a HDR donor template platforms.

## Clinical Translation of Gene Editing

With novel biological discoveries, there is no doubt that the gene editing field is flourishing. Good efficiencies and increased safety have contributed to its exponential development, and today the first gene editing-based clinical trials are underway. To date, more than 30 gene editing clinical trials have been approved, some of them incorporating CRISPR/Cas9 technology, and at least thirteen of them aim to correct β-hemoglobinopathies (see Table 1), which clearly constitutes a milestone in the biomedical field.

**TABLE 1:** Gene editing clinical trials for hemoglobinopathies updated as of December 2021.

NCT Number	Title	Conditions	Gene editing approach	Sponsor/Collaborators
NCT03432364	A Study to Assess the Safety, Tolerability, and Efficacy of ST-400 for Treatment of Transfusion-Dependent Beta-thalassemia (TDT)	•β-Thalassemia	Reexpression γ-globin by ZFN to disrupt an enhancer of the *BCL11A* gene	Sangamo Therapeutics
NCT03653247	A Study to Assess the Safety, Tolerability, and Efficacy of BIVV003 for Autologous Hematopoietic Stem Cell Transplantation in Patients With Severe Sickle Cell Disease (PRECIZN-1)	• Sickle Cell Disease	Reexpression γ-globin by ZFN to disrupt an enhancer of the *BCL11A* gene	Bioverativ, a Sanofi company / Sangamo
NCT04925206	A Safety and Efficacy Study Evaluating ET-01 in Subjects With Transfusion Dependent β-Thalassaemia	• Transfusion Dependent β-Thalassaemia	Reexpression γ-globin by CRISPR-Cas9 targeting the *BCL11A* erythroid-specific enhancer	EdiGene Inc.
NCT03745287	A Safety and Efficacy Study Evaluating CTX001 in Subjects With Severe Sickle Cell Disease	• Sickle Cell Disease	Reexpression γ-globin by CRISPR-Cas9 targeting the *BCL11A* erythroid-specific enhancer	Vertex Pharmaceuticals Incorporated
NCT03655678	A Safety and Efficacy Study Evaluating CTX001 in Subjects With Transfusion-Dependent β-Thalassemia	•β-Thalassemia	Reexpression γ-globin by CRISPR-Cas9 targeting the *BCL11A* erythroid-specific enhancer	Vertex Pharmaceuticals Incorporated
NCT04208529	A Long-term Follow-up Study in Subjects Who Received CTX001	•β-Thalassemia • Thalassemia • Sickle Cell Disease	Reexpression γ-globin by CRISPR-Cas9 targeting the *BCL11A* erythroid-specific enhancer	Vertex Pharmaceuticals Incorporated CRISPR Therapeutics
NCT04443907	Study of Safety and Efficacy of Genome-edited Hematopoietic Stem and Progenitor Cells in Sickle Cell Disease (SCD)	• Sickle Cell Disease	Reexpression γ-globin by CRISPR-Cas9 targeting *BCL11A* gene	Novartis/Intellia
NCT04205435	β-globin Restored Autologous HSC in β-thalassemia Major Patients	•β-Thalassemia Major	Reexpression γ-globin by CRISPR-Cas9 targeting globin regulators	Bioray Laboratories PLA 923 Hospital
NCT04853576	EDIT-301 for Autologous HSCT in Subjects With Severe Sickle Cell Disease	• Sickle Cell Disease • Hemoglobinopathies	Reexpression γ-globin by Cas12a RNP targeting the *HBG1/2* promoters	Editas Medicine, Inc.
NCT04774536	Transplantation of Clustered Regularly Interspaced Short Palindromic Repeats Modified Hematopoietic Progenitor Stem Cells (CRISPR_SCD001) in Patients With Severe Sickle Cell Disease	• Sickle Cell Disease	Targeting SCD-causing mutation by CRISPR/Cas9-ssODN	University of California
NCT04819841	Gene Correction in Autologous CD34^+^ Hematopoietic Stem Cells (HbS to HbA) to Treat Severe Sickle Cell Disease	• Sickle Cell Disease	HDR correction of SCD-causing mutation by CRISPR/Cas9-rAAV6	Graphite Bio, Inc.
NCT03728322	iHSCs With the Gene Correction of HBB Intervent Subjests With β-thalassemia Mutations	• Thalassemia	Correction of β-thalassemia-causing mutation by HDR of CRISPR/Cas9-piggyBac transposons in iPSC, previous to their differentiation into iHSCs	Allife Medical Science and Technology Co., Ltd.
NCT03655678	A Safety and Efficacy Study Evaluating CTX001 in Subjects With Transfusion-Dependent β-Thalassemia	•β-Thalassemia	Reexpression γ-globin by CRISPR-Cas9 targeting the *BCL11A* erythroid-specific enhancer	Vertex Pharmaceuticals Incorporated

Since 1980, many groups have pursued the correction of these pathologies by means of gene therapy using lentiviral vectors. It has been extremely challenging, especially in hemoglobinopathies, due to the strict requirements in terms of tight regulation of the globins, whose expression is restricted to the erythroid lineage. Therefore, lineage specific promoters were required in lentiviral constructs designed for the treatment of β-thalassemia and SCD, and are currently being tested in several clinical trials (Bueren et al., 2020; Ferrari et al., 2021). Taking into account this limitation, gene editing is postulated as an adequate evolution of gene therapy, able to fulfill the need for the physiological control of the transgene.

As mentioned throughout this review, many groups are doing their best to find a therapeutic approach by means of different gene editing strategies such as HDR or NHEJ. Several clinical trials are currently open for the evaluation of the feasibility of the autologous transplant of genetically-edited HSPCs for the treatment of SCD and β-thalassemia following myeloablative conditioning of the patients (Figure 1 and Table 1). Nine of these clinical trials are based on the previously described strategy with the rationale of interrupting the regulatory sequence of the erythroid enhancer of the *BCL11A* gene using ZFN, CRISPR/Cas9 or Cas12a. As previously mentioned, this sequence is involved in the prevention of the activation of fetal globin in adults. This strategy might be applicable to patients with SCD or with transfusion-dependent (TDT) β-thalassemia. Six multicenter phase 1/2 clinical trials are open for the evaluation of gene-edited autologous HSPC products. Two of these trials (NCT03745287 and NCT03655678) are evaluating the same CRISPR/Cas9-based medicinal product (CTX001) for SCD and TDT β-thalassemia sponsored by Vertex Pharmaceuticals Inc. and CRISPR Therapeutics. NCT03745287 is a phase 1/2/3 clinical trial with an estimated enrollment of 45 patients who have a very well documented severe SCD diagnosis, at least two severe vaso-occlusive crisis events per year and eligible for autologous HSCT. Similarly, NCT03655678 is also a phase 1/2/3 which will enroll 45 TDT β-thalassemia patients who have been received at least 100 mL/kg/year or 10 or more units/year of packed RBC transfusions in the 2 years before being enrolled. Following a similar approach, there is another clinical trial sponsored by Novartis Pharmaceuticals in collaboration with Intellia Therapeutics for the treatment of SCD (NCT04443907). This study is focused on evaluating the safety and efficacy of two medicinal products (OTQ923 and HIX763), which are CRISPR/Cas9 gene edited autologous HSPCs from SCD patients. This study is a phase 1/2 clinical trial recruiting 30 participant who have a confirmed SCD diagnosis and have suffered from at least one severe SCD side effect, such vaso-occlusive pain crisis, acute chest syndrome, recurrent priapism or prior stroke, or who have received chronic transfusions or RBC alloimmunization. Additionally, two other clinical trials (NCT03432364 and NCT03653247) are studying a similar gene editing approach, however, this one is based on ZFN technology. Sangamo Therapeutics is sponsoring NCT03432364, a phase 1/2 clinical trial where ST-400 (infusion of ZFN-modified autologous HSPCs) safety, tolerability, and efficacy will be evaluated in 6 TDT β-thalassemia patients with eight or more RBC transfusion per year in the 2 years before being recruited. On the other hand, Bioverativ is assessing the safety, tolerability, and efficacy of BIVV003 for autologous HSPCT in eight patients with severe SCD.

The first results of some of these clinical trials have recently been released. The first preliminary results of NCT03432364, where ST-400 is assessed for TDT β-thalassemia, has shown no positive result, due to relatively low genome editing efficiency associated with poor HbF expression (Smith et al., 2019; Brusson and Miccio, 2021). However, the infusion of CRISPR-edited CD34^+^ cells using CTX001 in two patients (one with SCD and one with TDT β-thalassemia) (NCT03745287 and NCT03655678) had high levels of allelic editing in bone marrow (up to 80% edited cells) and blood (around 60% edited cells), total hemoglobin level around 14 g/dL, increases in fetal hemoglobin, transfusion independence, and elimination of vaso-occlusive episodes in the patient with SCD more than a year after treatment (Frangoul et al., 2021). These very promising results confirm the good clinical outcome of this gene editing strategy to treat SCD and β-thalassemia.

Graphite Bio has recently launched a new phase 1/2 clinical trial (NCT04819841) for the treatment of SCD by means of another gene editing strategy based on the correction of the E6V mutation using CRISPR/Cas9 and rAAV6 setting (Dever et al., 2016; Lattanzi et al., 2021). This study is a first-in-human to study the safety and efficacy of the GPH101 medicinal product. This product is expected to be tested in 15 participants diagnosed with severe SCD who also suffer recurrent severe vaso-occlusive episodes and acute chest syndrome. In comparison with the other SCD trials previously mentioned, this one aims to directly correct the pathogenic sickle mutation in the *HBB* gene, thus preserving physiologic regulation of gene expression. This correction of the gene will also remove the expression of pathologic HbS, which will address the problem of competition with the highly expressed pathogenic protein (Lattanzi et al., 2021).

Apart from hemoglobinopathies, we and others are working on the generation of the required preclinical data that most likely will be translated into clinical trials in the near future (Fañanas-Baquero et al., 2021). Recently, our development based on CRISPR/Cas9 and rAAV6 for PKD has been granted Orphan Grant Designation by European Medicines Agency (EMA/OD/0000072308), which opens the path to future gene editing-based therapy for PKD patients (Figure 1). Gene editing for PKD patients will be a step forward in the therapy of this disease, for which there are currently only palliative treatments. Although new drugs are been evaluated, such as the allosteric activator of RPK, Mitapivat (Grace et al., 2019), autologous HSCT of gene edited HSPCs will represent the definitive therapy for PKD, since a single intervention will be able to correct the disease.

## Future Perspectives

Preclinical results and early clinical data have demonstrated the feasibility of genetically correcting hematopoietic and non-hematopoietic inherited diseases by *ex vivo* as well as *in vivo* strategies (Hoban et al., 2015, 2016; Dever et al., 2016; DeWitt et al., 2016; Ye et al., 2016; De Ravin et al., 2017; Koblan et al., 2021; Li et al., 2021). The current gene editing systems have started to move to the clinic to correct inherited RBC diseases. As of December 2021, there are 13 ongoing clinical trials for inherited RBC diseases (Table 1). The three main bottlenecks of these innovative therapies are: (i) increasing efficacy to target long-term HSPCs, (ii) assessing safety and, (iii) facilitating patient access to gene editing therapies. This last point is even more important in the case of the hemoglobinopathies due to the large number of affected patients. Although gene editing for RBC diseases is already benefiting hemoglobinopathy patients (Frangoul et al., 2021), this promising therapy has to be more accessible to treat severe patients worldwide.

The available gene editing tools and their use in different approaches can achieve good efficiency with adequate safety in the treatment of inherited RBC diseases. However, gene therapy will be considered a real therapeutic option for these patients only when the production of high numbers of adequately corrected cell products is not challenging. This is more relevant in the case of strategies based on HDR. The development of new modified components involved in the HDR process could increase HDR efficiency and facilitate its potential application to a wider range of genetic diseases. Different strategies are now being tested. Base editors are directed to single point mutations that would require one specific development for each gene mutation. Prime editing is focused on the use of longer gRNAs that carry not only the genomic recognition site, but also the genetic material to facilitate the correction of the mutated sequence. Experimental evidence points to the co-localization of the different components required for gene editing (nucleases, donor templates, additional enhancer proteins, etc.) to facilitate the process and increase efficiency. Thus, new developments to promote this subcellular co-localization are required to increase the potential application of therapeutic gene editing.

On the other hand, the delivery of the gene editing components into the cell and more importantly, into the cell nucleus, while avoiding degradation by endogenous cellular mechanisms in that journey, is a key factor for gene editing. In ongoing preclinical developments (Dever et al., 2016; Schiroli et al., 2019; De Ravin et al., 2021; Fañanas-Baquero et al., 2021; Wilkinson et al., 2021) and in recently approved clinical trials for the treatment of SCD (see Table 1), electroporation and AAVs are used to deliver the endonuclease ribonucleoprotein and the donor DNA template, respectively. Electroporation has intrinsic toxicity and AAVs require highly complex developments in their manufacturing and imply significant costs. Less toxic, easier and, if possible, less expensive procedures would be required to facilitate the development of these therapeutic strategies. Alternative delivery systems, such as nanoparticles (Burns, 2021) or virus-like particles (Mangeot et al., 2019; Hamilton et al., 2021), together with new donors (Shy et al., 2021) will simplify the manufacturing process, thus making them more accessible to more patients.

In the meantime, HSCT of *ex vivo* gene edited cells is still a limiting factor for the correction of inherited hematopoietic diseases. This is even more relevant in diseases where the required percentage of corrected long-term HSPCs engrafted is high or when there is no selective advantage of the corrected over the non-corrected ones. In this scenario, a conditioning regimen is required to eliminate the endogenous diseased cells, which could compete against the corrected cells infused after the *ex vivo* editing process. These conditioning regimens are highly genotoxic and are associated with immunosuppression, tissue damage and even sterility. Again, new approaches need to be explored to develop non-genotoxic conditioning regimens that would allow sufficient engraftment of corrected healthy cells to achieve a therapeutic effect.

Furthermore, the potential use of *in vivo* delivery methods would facilitate addressing genetic diseases affecting the hematopoietic system itself or even other organs/systems, which would not be possible to manipulate for *ex vivo* correction, such as liver, kidney, lung or brain. Consequently, development of novel recombinant virus-based systems or nanoparticle approaches targeting precise tissues or very well defined cell populations, such as HSPCs, will facilitate the use of gene editing without complex cell manipulation processes. However, tight control of the off-target tissue effect will need to be kept to reduce side effects of delivering gene editing tools into patient organs.

Altogether, we are at the beginning of a rapidly changing field, where new gene editing tools and approaches are appearing constantly. Gene editing is opening new therapeutic opportunities to treat patients suffering inherited RBC diseases. However, making this technology available to a great number of patients remains a challenge.

## Author Contributions

J-CS and OQ-B designed, organized, wrote and reviewed the manuscript. SF-B, MD-R, and IO-P wrote the sections and reviewed the manuscript. All authors contributed to the article and approved the submitted version.

## Conflict of Interest

J-CS is a consultant for Rocket Pharmaceuticals. The remaining authors declare that the research was conducted in the absence of any commercial or financial relationships that could be construed as a potential conflict of interest. The handling editor declared a past co-authorship with one of the authors.

## Publisher’s Note

All claims expressed in this article are solely those of the authors and do not necessarily represent those of their affiliated organizations, or those of the publisher, the editors and the reviewers. Any product that may be evaluated in this article, or claim that may be made by its manufacturer, is not guaranteed or endorsed by the publisher.

## Footnotes

1
https://bit.ly/3DDJ4X4

2
https://bit.ly/3fRSunf
